# The Effect of Transcutaneous Auricular Vagal Nerve Stimulation (taVNS) on P3 Event-Related Potentials during a Bayesian Oddball Task

**DOI:** 10.3390/brainsci10060404

**Published:** 2020-06-25

**Authors:** Claire V. Warren, María J. Maraver, Alberto de Luca, Bruno Kopp

**Affiliations:** 1 Clinic of Neurology, Hannover Medical School, 30519 Hannover, Germany; kopp.bruno@mh-hannover.de; 2 Institute of Psychology, Leiden University, 2333 Leiden, The Netherlands; mjmaraver@psicologia.ulisboa.pt (M.J.M.); a.de.luca@fsw.leidenuniv.nl (A.d.L.); 3 Faculty of Psychology, University of Lisbon, 1649-013 Lisbon, Portugal

**Keywords:** norepinephrine, event-related potentials, transcutaneous auricular vagal nerve stimulation, P300, oddball, neuromodulation

## Abstract

Transcutaneous auricular Vagal Nerve Stimulation (taVNS) is a non-invasive brain stimulation technique associated with possible modulation of norepinephrinergic (NE) activity. NE is suspected to contribute to generation of the P3 event-related potential. Recent evidence has produced equivocal evidence whether taVNS influences the P3 in healthy individuals during oddball tasks. We examined the effect of taVNS on P3 amplitudes using a novel visual Bayesian oddball task, which presented 200 sequences of three stimuli. The three consecutive stimuli in each sequence are labelled Draw 1, Draw 2 and Draw 3. In total, 47 Subjects completed this visual Bayesian oddball task under randomised sham and active taVNS stimulation in parallel with an electroencephalographic (EEG) recording. We conducted exploratory analyses of the effect of taVNS on P3 amplitudes separately for Draws. We found typical oddball effects on P3 amplitudes at Draws 1 and 2, but not Draw 3. At Draw 2, the oddball effect was enhanced during active compared to sham taVNS stimulation. These data provide evidence that taVNS influences parietal P3 amplitudes under specific circumstances. Only P3 amplitudes at Draw 2 were affected, which may relate to closure of Bayesian inference after Draw 2. Our findings seemingly support previously reported links between taVNS and the NE system.

## 1. Introduction

The vagus nerve is an autonomic nerve which regulates major organs and physiological responses [1]. Invasive vagal nerve stimulation (VNS) has been used as a treatment for disorders such as epilepsy and depression [2,3,4,5], the success of which has been attributed to possible activation of the Locus Coeruleus norepinephrinergic system (LC-NE) [6,7,8]. The LC is innervated by the solitary tract, a brainstem nucleus for vagus nerve afferents [9,10] and lesions to this region abolish the therapeutic effects of VNS in both depression and epilepsy [11,12,13]. Furthermore, VNS directly increased NE concentration in rats [14,15,16], and it progressively increased the basal firing rate of LC NE neurons with long-term VNS treatment [17].

Transcutaneous auricular vagal nerve stimulation (taVNS) is a new form of supposed brain stimulation. It is theorized to target the LC in a similar way to VNS, though using non-invasive methods. The auricular branch of the vagus nerve supplies the cymba conchae (i.e., the inner part of the auricle [18,19]), as well as the tragus [20,21,22]. Active stimulation to the skin at these sites is suspected to activate the auricular vagus nerve, which has fibers projecting to the nucleus tractus solitarius (NTS) [23]. The NTS is connected to additional structures in the brainstem, including the LC [24]. Active taVNS has been shown to increase cortical excitability [25], while others have reported comparable fMRI activity after both VNS and taVNS [26]. Active taVNS stimulation in humans has shown indicators of physiological and hormonal NE activation, such as increased salivary alpha amylase levels [27,28,29]. Human behavioural studies have also demonstrated that taVNS effectively manipulates some behaviours associated with the LC-NE system, such as post-error slowing [30] and action cascading [31]. Sequential modulation during the Simon task (which evaluates adaptation to location-based response conflict, also related to NE) [29] was more pronounced under active taVNS, which manifested as an enhanced reduction in reaction times (RTs) in incompatible trials compared to compatible. However, because the field is relatively new there is great heterogeneity in the stimulation parameters used across studies and the exact mechanisms of taVNS are yet not fully understood. Besides that, taVNS has already shown promise as a potential brain stimulation method that may modulate cognitive processes related to activity in the NE system of the brain.

One of the many benefits of taVNS as a potential method of brain stimulation is its flexible nature. The device is compact, portable and requires low maintenance. This allows it to be used in various formats and in combination with many forms of brain imaging with little chance of interference. For example, electroencephalography (EEG), and the study of event-related-potentials (ERPs) in particular, allows to examine brain activity efficiently and non-invasively. The majority of taVNS-EEG studies focused on the parietally-distributed P3b, presumably due to the suggested shared links with NE. The P3b is thought to be affected by the LC-NE system [32]. Furthermore, links between phasic pupil dilations, an indicator of LC activity and P3 amplitudes have been reported [33].

**Figure 1:**
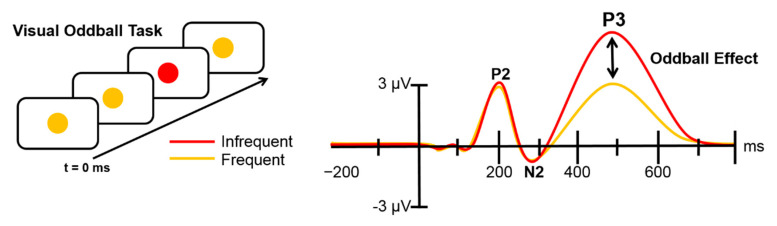
A basic visual oddball task and exemplary amplitudes that it elicits. The P3 for infrequent stimuli is notable larger than the P3 for frequent stimuli. Also included are the P2 and N2 components [38].

The P3 is a large positive deflection, with onset approximately 300 ms after the presentation of a stimulus, which may be in auditory or visual modality [34]. It reflects the orienting response towards the eliciting event and can be easily demonstrated using an oddball task, in which stimuli occur at varying degrees of frequency ([35]; Figure 1). In 2-stimulus oddball tasks, one stimulus (the Standard) appears at a much higher frequency than the other (the Target). During a typical active (or ‘attended’) oddball task, subjects must provide a button response when the Target appears [36], as opposed to a passive (or ‘ignored’) oddball task, in which no response is required. The Target typically elicits larger P3 amplitudes than the Standard, i.e., the Oddball Effect [37].

As VNS has many applications, it would be of interest to discover whether the non-invasive variant of taVNS has similar brain stimulation effects. The P3 is already used as an indicator of sensitivity to VNS in epilepsy [39] and both VNS and taVNS have been linked to the LC-NE [8,11,12,13,18]. Few studies have investigated the effect of taVNS stimulation on the parietally-distributed P3b component during oddball tasks in healthy subjects, but their results remain inconclusive. A larger P3b during active taVNS stimulation compared to sham stimulation has been reported [40]. Another study found larger P3b amplitudes for easy targets during active taVNS stimulation, but not for difficult targets [41]. In three separate experiments, one report failed to find a significant effect of active taVNS stimulation on P3b amplitudes in various auditory and visual oddball tasks [42]. No effect of taVNS on P3b amplitudes was found when using a version of the Simon task, evaluating adaptation to location-based response conflict [29]. However, N2 amplitude attenuation was increased for conflict trials during the active condition. This small pool of studies provides limited insight into the mechanisms of taVNS. Two studies did not find a significant effect of taVNS on oddball P3b amplitudes [29,42]. However, the two studies that returned significant effects did so in specific circumstances during particular versions of the oddball task [40,41]. Ultimately, the literature fails to reach a conclusion on the influence of taVNS on the P3 during oddball tasks, leaving open questions for further research.

**Figure 2:**
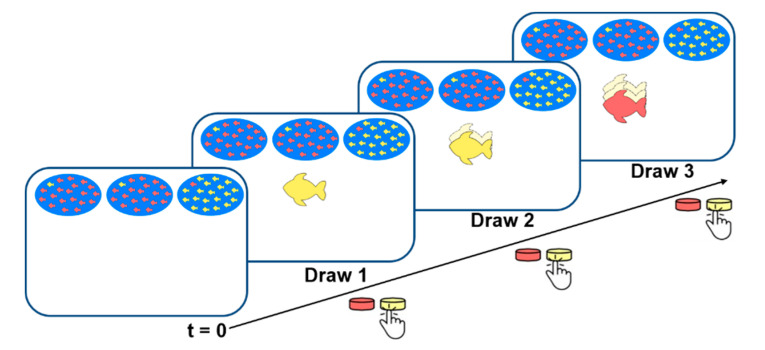
An example of a sequence in the Bayesian oddball task [40]. Before stimulus presentation, the DL is twice as likely as a SL. In the following sequence, the subject sees a SLF at Draw 1, and indicates that they believe the SL to be more likely. A faded colour and broken outline indicate that this fish has been returned to the lake before the next fish is drawn. In this case, a SLF was presented at Draw 2, followed by a SL selection from the participant as indicated by the button press. Finally, a DLF fish is shown and the participant still believes the SL to be more likely.

Assuming that taVNS influences NE activation similarly to VNS, and given the evidence suggesting that parietal P3 amplitudes are affected by the LC-NE system [32], we hypothesized that taVNS may in fact affect the P3 during oddball tasks. We suggest that the traditional oddball task is not sensitive enough to render the particular brain stimulation effects of taVNS detectable. With the aim to overcome this methodological limitation, we created a Bayesian oddball task (Figure 2); a variant of the traditional oddball paradigm that can track the Bayesian beliefs of the subjects regarding stimulus probability, as well as more specific sequential information [43]. Sequential effects of the oddball task on P3 amplitudes are not usually investigated as trials are typically averaged together following task completion. The Bayesian oddball task [44] resembles an active, rather than passive, oddball task as participants are required to respond to every stimulus. Subjects must consider the stimulus as a sample drawn from one of two populations, and to think about its most probable origin.

Similar to traditional oddball tasks, the Bayesian oddball task [44] has two stimuli: one frequent and one infrequent. In this task, the likelihood of these stimuli is made known to the subjects; this information is not available in traditional oddball tasks. In our particular version of the Bayesian oddball task, the participant is shown 3 lakes containing opposite distributions of the stimuli. Two lakes (i.e., Double Lakes) contain predominantly red fish (Double Lake Fish; DLF). One Lake (i.e., Single Lake) contains mostly yellow fish (Single Lake Fish; SLF). The DLF and SLF resemble the Standard and Target stimuli of a traditional oddball paradigm, respectively. Participants are told that a random lake has been chosen and that a sequence of three fish will be drawn from the selected lake. After each fish presentation, subjects must indicate on a keypad which lake they suspect to have been randomly chosen, based on the perceived probability of the sequence drawn. The learning that occurs at each step is called “Bayesian inference”. Subjects have an initial or “prior” hypothesis about the probability that a particular lake type has been selected, based on its prevalence. This may change after seeing the evidence of each fish (at Draws 1, 2 and 3), thus becoming updated to the “posterior” hypothesis formed from the integration of the new knowledge, which then serves as a new “prior” hypothesis. Detailed descriptions of this Bayesian inference process can be found in previous publications from our group [44,45,46,47,48,49]. Each fish is returned to the lake after a response is given, ensuring that the proportions of fish in each lake remains the same. The benefit of this task is that it allows us to numerically calculate the Bayesian surprise of each stimulus, which are linked to P3 amplitude [44]. Therefore, the subjects’ posterior hypotheses following each Draw can also be calculated and manipulated. Other tasks have manipulated the P3b using hard versus easy targets [41], though the differences between conditions are not quantifiable.

Given the limited nature of the literature, we developed a particular version of the Bayesian oddball task [44] to provide clarity on mechanisms of taVNS, using more specific situations under which the P3 can be observed. taVNS may have value as a method of brain stimulation, as its non-invasive nature and quick application make it an ideal tool for use in behavioural studies or potential therapies. Similarly, the taVNS device can be used in tandem with EEG, placing it at a potential advantage to other methods of brain stimulation. Our aim was therefore to conduct an exploratory analysis to examine the neuromodulatory effects of taVNS on the P3 of healthy young subjects during our novel Bayesian oddball task. In the case that taVNS is a valid form of brain stimulation, we expected to find augmenting effects of taVNS on P3 amplitudes.

## 2. Experimental Section

### 2.1. Sample

Forty-seven students were recruited from the University of Leiden. Exclusion criteria were a history of neurological or psychiatric conditions, current pregnancy, or claustrophobia. Of the original *N =* 47 subjects, *n =* 1 subject was excluded from further analysis for not attending their second session. An additional *n =* 4 were removed from the study due to technical issues with the EEG equipment.

The mean age of the remaining sample (*N =* 42; 8 male) was 20.55 years (*SD =* 2.18, range = 18–25). The mean number of days between the two recording sessions was 12.88 days (*SD =* 7.27, range = 7–35). Written informed consent was obtained from each subject. The experiment conformed to the ethical standards of the Declaration of Helsinki (World Health Organisation, 2013) and the protocol was approved by the local ethics committee (Leiden University, Institute for Psychological Research).

### 2.2. Materials and Procedures

Each participant completed 2 sessions (each separated by a minimum of 7 days); one whilst undergoing the active taVNS condition, and the other while receiving the sham taVNS condition. The order of the taVNS conditions was counterbalanced across subjects consistent with previously published protocols [50,51,52,53,54]. The first session began with a battery of forms and screening questionnaires, consistently used in previous protocols [50,51,52,53,54] to control for recent use of psychedelic drugs, use of psychiatric medication, or caffeine consumption in the 3 h preceding the recording session. Following this, subjects were seated in the EEG sound-proof booth and the taVNS device was applied. In order to ensure that stimulation had taken effect at the start of the experiment, stimulation was operational for a minimum of 15 min before the behavioural paradigm began and continued until the end of the task [27]. Subjects then completed the Bayesian oddball behavioural task whilst undergoing an EEG recording. The second session was identical with the exception of the questionnaires prior to recording.

### 2.3. The Bayesian Oddball Paradigm

**Figure 3:**
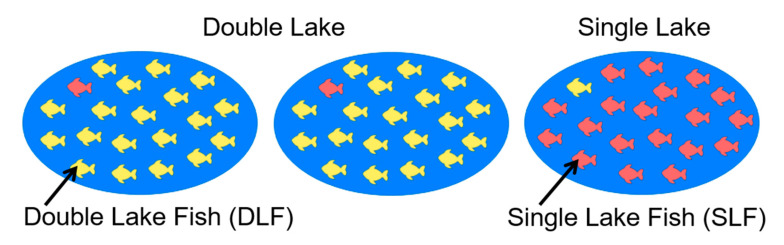
Terminology and stimuli used in the particular version of the Bayesian oddball task [44].

In the task, subjects are first shown a selection of lakes which contain opposite proportions of red and yellow fish. The Double Lakes (DL; Figure 3) contained 95% red fish (double-lake fish; DLF) and 5% yellow fish (single-lake fish; SLF). The Single Lake (SL) contained the opposite proportions. Subjects were told that one of the lakes had been randomly selected and that 3 fish would be drawn consecutively from this chosen lake. Each fish would be returned to the lake before the following was drawn in order to maintain the proportions. The subjects’ task was to indicate on a keypad whether they believed the DL or SL to be more likely, based on the fish that were drawn. Subjects received one of four versions of the task which balanced the left/right orientation of the DL and SL and alternated the dominant colour from red to yellow (see Table A1). Each stimulus (fish) was presented 500 ms after the previous response had been given. The paradigm consisted of 200 sequences, with two opportunities for short rests. Providing subjects with the lakes, and therefore the prior probability of each lake, allows us to calculate the numeric value of Bayesian surprise, which have previously been linked to P3b amplitude [44]. The optimal prior probabilities for this task (i.e., the DL/SL ratio) have been selected based on the Bayesian surprise calculated from Bayes’ Theorem ([43]; see Table A2). We therefore chose a design with an extreme contrast in the prior probability for the SL and DL, in order to create distinct amplitude differences for DLF and SLF. This was also true for our examination of predicted peak amplitude differences for consecutive fish in each sequence (Draw 1, Draw 2, Draw 3). Each of the 8 possible sequences was presented at the relative frequency determined by Bayesian probability.

The novel behavioural measure of this task is the percentage of Double Lake Selection for each stimulus at each Draw. This is defined as the percentage of responses which selected the Double Lake from the total number of sequences. This can be expressed by the following:

RTs from each stimulus-locked response were also recorded, though participants were told that accuracy was more important than speed.

$$\%\mathrm{DLS}=\frac{N\;\mathrm{Double}\;\mathrm{Lakes}\;\mathrm{Selections}}{N\;\mathrm{Total}\;\mathrm{Lake}\;\mathrm{Selections}}\times 100$$

### 2.4. taVNS Stimulation

A NEMOS^®^ taVNS device was used. The earpiece composed of 2 titan electrodes mounted on a moldable gel frame. These electrodes projected electrical pulses onto the surface of the skin via an electrical battery pack. For the active condition, the earpiece was always inserted into the left ear, according to standard research convention [55] and both electrodes made contact with the cymbae concha. For the sham stimulation, the earpiece was inserted into the auricle upside-down, so that the electrodes made contact with the earlobe, which has been shown to be relatively free of vagal afferents [56,57]. The device was turned on and activated in both conditions, with typical 30 s periods of activity followed by 30 s of inactivity, until the end of the behavioural experiment. Stimulation intensity was set to 0.5 mA, with pulses every 200–300 ms, at a frequency of 25 Hz. These stimulation parameters were chosen in correspondence with previously recommended guidelines [20], and are consistent with previously published experimental designs [31,50,51,52,53,54].

### 2.5. Electrophysiological Data Recording

EEG recordings took place in a shielded, sound-proofed 8 m^2^ booth. Patients were seated 150 cm from the screen in an unadjustable chair. Continuous unfiltered raw data were recorded using a 32 channel standard Quickamp amplifier. The 32 Ag/AgCl ring electrodes were arranged according to the extended international 10/20-System on a BioSemi headcap (Birmingham, United Kingdom) with an adjustable chinstrap. The recording software was ActiView 7.05 (BioSemi, Birmingham, United Kingdom). The sampling rate was 256 Hz (high-pass filter: 0.16 Hz low-pass filter: 100 Hz). The Common Mode Sense (CMS) was the active electrode and Driven Right Leg (DRL) was used as the passive electrode. Electrode impedance was kept below 20 kΩ. Vertical and horizontal electro-oculograms were recorded using two electrodes positioned at the left and right suborbital ridges and two electrodes above and below the external ocular canthus of the left eye to monitor ocular artefacts.

EEG data were analysed offline using BrainVision Analyzer 2.0 (Brain Products, Gilching, Germany). A low-pass filter of 70 Hz was applied, as well as a notch filter of 50 HZ to reduce electrical noise. Through visual inspection, the 25 Hz taVNS pulses were visible in the left-lateralized electrodes for some subjects. To counter this problem, a 25 Hz Band Width filter (similar to the above notch filter) was applied to all recordings (order 4). ICA was used to remove ocular artefacts from the data, which were then screened for artefacts (voltage step > 75μV/ms; low activity 0.5 μV/100 ms; activity 150 μV/200 ms; max/min amplitude +/−100 μV) and then rejected. Data were segmented according to the DLF and SLF for each of the 3 Draws (DLF 1, SLF 1, DLF 2, SLF 2, DLF 3, SLF 3) and baseline corrected to 200 ms pre-stimulus. The average reference was used. In addition to this typical pre-processing procedure, we applied the additional step of Residue Iteration Composition (RIDE) [58] to address the common problem of individual ERP latency jitter. Segmented (un-averaged) trial-by-trial data were exported to MATLAB (R2018a), where individual subjects’ grand averages were formed using RIDE. Table A3 displays the numbers of epochs included in the ERP analyses for each subject.

### 2.6. Statistical Analysis

The %DLS and RTs were calculated for each stimulus at each Draw as described in the introduction. Oddball behavioural data were not the primary focus of the Bayesian oddball task [40]. Therefore, inferential statistics were not performed and behavioural data serve descriptive purposes only. Further information on %DLS and RTs can be found in Appendix D.

For EEG data, an appropriate time window needed to be selected. Based on visual inspection of a left-lateral shift, which is somewhat atypical compared to the literature [59], we chose to inspect the lateral parietal electrodes (P3, Pz, P4) at the apparent distribution of the P3b during 300–500 ms. We subsequently divided this time window into the following 50 ms increments: (300–349 ms), (350–499 ms), (400–449 ms) and (450–499 ms).

We conducted a 2 × 2 × 3 Repeated Measures ANOVA separately for each Draw, using the factors Stimulus (DLF vs. SLF), taVNS (Active vs. Sham), and Electrode (P3 vs. Pz vs. P4) as within-subject factors.

We define the *Oddball Effect* as a main effect of Stimulus. This is the difference in amplitudes between the frequent and infrequent stimuli in each condition i.e., the DLF amplitudes subtracted from the SLF.

The *taVNS-Oddball Effect* is the difference between the *Oddball Effect* in the active and sham conditions (i.e., a taVNS × Stimulus interaction). To calculate this, we obtained the difference waves for each Draw in the active condition i.e., the *Oddball Effect*. We then did the same for each Draw at the sham condition. Finally, we subtracted these sets of difference waves and produced a new set of difference waves, representing the complete effect of active taVNS stimulation at each Draw compared to sham stimulation. Simplified, the *taVNS-Oddball Effect is*:

(SLF active−DLF active)−(SLF sham−DLF sham)

Or

(*Oddball Effect* in the Active Condition)−(*Oddball Effect* in the Sham Condition)

We used a conservative significance level of α = 0.01. For ANOVAs, η_p_^2^ (partial eta squared) is used to indicate effect size. Sphericity was not assumed; therefore, the results were reported using the Greenhouse–Geisser method.

## 3. Results

### 3.1. Behavioural Data

#### 3.1.1. %DLS

**Figure A1:**
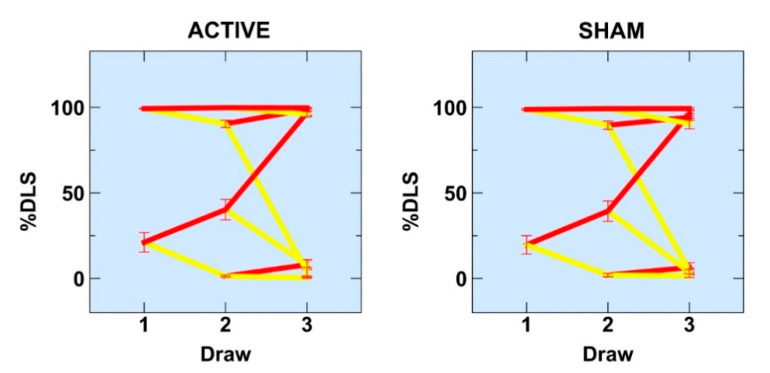
The mean percentage Double Lake Selection (%DLS) for both the active and sham conditions at each Draw of the 8 possible Sequences. Continuous red lines indicate a DLF, while yellow lines represent an SLF.

In over 95% of the sequences presented, the participants appeared to reach a final decision or “Lake Conclusion” at Draw 2. From the descriptive data, it appears that subjects did not change their decision following their choice at Draw 2 and the stimuli presented at Draw 3 were then inconsequential for the following selections. This is also consistent with Bayesian expectations. Further information regarding the sequence probability distribution, %DLS and RTs can be found in the Figure A1 and Table A4.

#### 3.1.2. ERP Analysis

**Figure 4:**
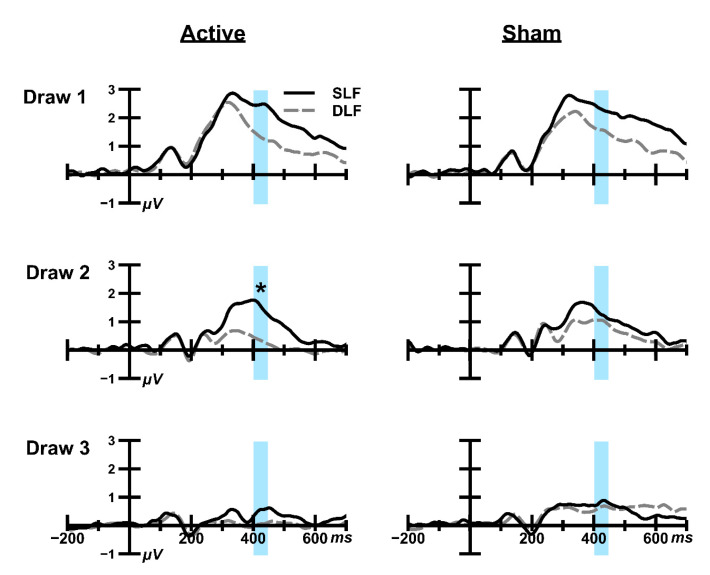
The Oddball Effect at each Draw during active and sham stimulation at electrode P3. The analysed time window of 400–449 ms has been highlighted. The taVNS-Oddball Effect is the difference between the Oddball Effect for active and sham taVNS stimulation in these highlighted areas. The effect was most visible at the P3 electrode (displayed), although this was not statistically significantly different from the Pz or P4 electrodes. Data have been high-pass filtered (12 Hz, Order 2) for display purposes only. * The interaction between taVNS and P3 amplitudes was statistically significant at Draw 2 (*p* < 0.01), but not at Draw 1 (*p* = 0.68) or Draw 3 (*p* = 0.91).

We report the findings from the time-window of 400 ms–449 ms (Figure 4), as this returned the most interesting effects of taVNS from inferential statistical analysis and previous studies also focused on similar epochs for active oddball variants [60,61]. Analyses of the other time windows can be found in the Appendix E.

##### Draw 1

A typical main effect of Stimulus was found at the parietal electrodes (*F*(1,41) = 25.60, *p* < 0.001, η***_p_***^2^ = 0.42), with further inspection confirming that the SLF produced larger amplitudes than the DLF. This is consistent with the oddball effect reported in traditional paradigms [62,63]. The main effect of taVNS was not significant (*F*(1,41) = 0.29, *p* = 0.60, η***_p_***^2^ = 0.01) and there was no interaction between taVNS and Stimulus (*F*(1,42) = 0.17, *p* = 0.68, η***_p_***^2^ < 0.01).

##### Draw 2

A main effect of Stimulus was found at Draw 2 (*F*(1,41) = 26.61, *p* < 0.001, η***_p_***^2^ = 0.39). The main effect of taVNS was not significant (*F*(1,41) = 0.02, *p* = 0.90, η***_p_***^2^ < 0.01). The interaction between taVNS × Stimulus × Electrode was also not statistically significant (*F*(2,82) = 1.26, *p* = 0.29, η***_p_***^2^ = 0.03). The taVNS × Stimulus interaction (i.e., *taVNS-Oddball Effect)* was statistically significant (*F*(1,41) = 11.66, *p* < 0.01, η***_p_***^2^ = 0.22), in which the oddball effect during active stimulation was larger than during sham stimulation (Figure 4). To investigate this interaction further, a series of post-hoc tests were conducted.

We first employed paired samples t-tests to examine the oddball effect in each session, i.e., the difference between DLF and SLF amplitudes. During the active condition, we found a statistically significant oddball effect for each of the parietal electrodes (P3: *t*(41) = −4.79, *p* < 0.000; *d* = 0.738; Pz: *t*(41) = −4.02, *p* < 0.000; *d* = 0.617 P4: *t*(41) = −3.29, *p* < 0.01; *d* = 0.509). In the sham condition no statistically significant effects were found (P3: *t*(41) = −0.76, *p* = 0.45; *d* = 0.118; Pz: *t*(41) = −1.35, *p* = 0.18; *d* = 0.210; P4: *t*(41) = −0.74, *p* = 0.46; *d* = 0.115).

Next, we used paired sampled t-tests to examine how each stimulus was affected by taVNS stimulation. We compared DFL amplitudes during active stimulation compared to sham stimulation. This revealed no statistically significant effects (P3: *t*(41) = −1.82, *p* = 0.08; *d* = 0.278; Pz: *t*(41) = −1.57, *p* = 0.12; *d* = 0.241; P4: *t*(41) = −1.24, *p* = 0.22; *d* = 0.190). SLF amplitudes were then compared between active and sham stimulation, and similarly, no statistically significant effects were found (P3: *t*(41) = 2.02, *p* = 0.05; *d* = 0.313; Pz: *t*(41) = 0.68, *p* = 0.50; *d* = 0.103; P4: *t*(41) = 0.79, *p* = 0.43; *d* = 0.124).

Posterior Bayesian probabilities (Table A2 in Appendix B) inform us that decisions reach >99% certainty following 2 consecutive identical stimuli, i.e., SLF-SLF or DLF-DLF. When these DLF-DLF and SLF-SLF sequences were exclusively analysed, the taVNS × Stimulus interaction remained significant (*F*(1,41) = 9.80, *p* < 0.01, η***_p_***^2^ = 0.19).

##### Draw 3

No main effect of Stimulus (*F*(1,41) = 1.86, *p* = 0.18, η***_p_***^2^ = 0.04) or taVNS (*F*(1,41) = 0.74, *p* = 0.39, η***_p_***^2^ = 0.02) was present at Draw 3; a novel finding in oddball research. The interaction between taVNS and Stimulus was not statistically significant (*F*(1,41) = 0.01, *p* = 0.91, η***_p_***^2^ < 0.01).

## 4. Discussion

In this study, we examined the effect of taVNS stimulation on P3 amplitudes using a novel Bayesian oddball task [44]. This Bayesian oddball task [44] was chosen as a more sensitive alternative to typical task variants, exploring some of the possible reasons for the inconsistent reports of taVNS on P3 amplitudes. Earlier studies using taVNS to study the P3 suggested that taVNS was sensitive to particular circumstances under which the P3 was produced. The Bayesian oddball task has the methodological advantage of distinguishing sequential effects of oddball stimuli on P3 amplitudes.

Although the behavioral data were only used for descriptive purposes, the %DLS appeared not to change from Draw 2 to Draw 3 (Table A2). We suggest that our subjects came to a definite conclusion at Draw 2 following 2 consecutive identical stimuli (i.e., fish colors), after which, new information was no longer used for decision-making. We refer to this Bayesian inference process as ‘Lake Conclusion’.

Consistent with typical oddball tasks, a statistically significant *Oddball Effect* was seen on P3 amplitudes at Draw 1 and Draw 2. However, this was not present at Draw 3. We primarily report a statistically significant medium-sized *taVNS-Oddball Effect* on the P3 at the parietal electrodes but only during Draw 2 during the time window of 400–449 ms.

Other available literature has also reported effects of taVNS on specific aspects of neural brain correlates. One study reported enlarged P3 amplitudes in response to infrequent stimuli during active stimulation in an oddball task [40]. Another study found an enhanced parietal P3 during active taVNS stimulation [41], although other report failed to replicate these results [42]. This failure to replicate may be due to the sensitivity of taVNS to the paradigm, as the previous study used a variety of stimuli and reported the effect on only easy (but not difficult) stimuli [41], whereas the later study used a typical simple variant of a 2-stimulus oddball task [42]. During an adapted Simon task, an enhanced attenuation of N2 amplitude after conflict during active taVNS stimulation was reported [29]. Our results appear to concur with the majority of the previous literature which suggests that taVNS is a valid tool of brain stimulation, though in specific circumstances only. We suggest that the Bayesian probabilities of stimuli in a task may be related to the specific circumstances, which influence the properties of the interaction between taVNS stimulation and the cognitive demands of the task at hand. Our results support this suggestion, as the taVNS effect was only visible at Draw 2, where the Bayesian surprise was largest and participants seemed to complete their final decision. Similarly, a previous study showed an effect of taVNS when the stimuli were easy to distinguish, but not when the distinction was unclear [41], although this was not quantifiable from a Bayesian perspective.

Our Bayesian oddball paradigm [44] is a 2-stimulus active oddball variant that allows us to examine more specific neurocognitive processes compared to the traditional oddball literature. Traditional oddball protocol averages all trials with equal weight and thus, sequential nuance may be lost. Other studies have reported a gradual decline in P3 amplitude (i.e., habituation) during the course of the task [64]. We used the Bayesian oddball task [44] to investigate the effect of oddball sequences. Due to this sequential tracking, our task also allows us to track the subjects’ probabilistic beliefs during the course of the task. Unlike a traditional oddball paradigm, this Bayesian oddball task requires the subject to make a probabilistic decision after each Draw, which should indicate their current hypothesis about which lake type is more probable (DL or SL). This belief updating can be quantified from a Bayesian perspective (Table A2). That is, we can identify when a subject could reach a Lake Conclusion and requires no more information to finalise their belief.

According to Bayesian posterior probabilities (Table A2), the majority of task-relevant information for a Lake Conclusion is received at Draws 1 and 2 of the Bayesian oddball task. More specifically, this occurred when the two consecutive stimuli were identical (SLF-SLF or DLF-DLF), which occurred in 90.5% of the initial two stimulus sequences. This may explain the presence of a significant *Oddball Effect* at Draws 1 and 2 but not at Draw 3. The importance of the stimuli at Draw 3 may be minimised after a Lake Conclusion has already been formed at Draw 2. This interpretation can be supported by Desmedt (1980), who suggests that the P3 following a response reflects a “post-decision closure” [65], rather than a pre-decision process. Desmedt postulates that closure events reflect the optimization of organizational behaviour and attentional resources in response with task demands [65]. This post-decision closure may offer an explanation for the specific mechanisms present at Draw 2, which produced the *taVNS-Oddball* effect. This interpretation is also compatible with the apparent absence of the P3 at Draw 3, when a decision was no longer being made. Another study reported enhanced P3 amplitudes for a Target stimulus, but only when the preceding cue was a successful predictor of this stimulus [66]. In that case, subjects also appeared to reach “post-decision closure” once a prediction was resolved. A similar case may be present in our results, as the Lake Conclusion at Draw 2 reflects a closure event following a prediction.

The use of Lake and Fish in our paradigm was deliberately chosen to resemble a behavioural task used to examine Jumping to Conclusions (JTC) in patients with schizophrenia and psychosis [67,68,69,70,71]. Similar to our design, Speechley et al. (2010) also used Bayesian probabilities to design their paradigm and identify the most meaningful sequences [68]. These studies contained up to 10 Draws per sequence. Our design restricted sequences to 3 Draws. From a Bayesian perspective, there is little new information available after Draw 3. Sequences either reached 0.99 certainty after Draw 2 (e.g., DLF-DLF-DLF-DLF) or began to repeat information (e.g., DLF-SLF-DLF-SLF). Our restriction of 3 Draws per Sequence contained the most information and allowed us to record a higher number of trials overall. This study examined the electrophysiological correlates of a JTC task and we also report an apparent tendency of subjects to follow cumulative Bayesian reasoning during responding, rather than trial-by-trial evidence.

Previous studies of VNS in animals and humans, as well as physiological and behavioural investigations of taVNS have suggested a strong link between vagal nerve activation and the LC-NE [6,7,8,16,19,24,25]. This is suspected to be done by innervating an indirect pathway through the NTS and other structures in the brainstem, primarily the paragigantocellularis (PGi) [72]. A previous EEG investigation of taVNS suggests that taVNS-related behavioural and electrophysiological changes are influenced primarily by NE [29]. This is due to a number of animal studies showing that direct innervation of the vagal nerve activates the LC (through this indirect system in the brainstem). Furthermore, a review of the neural basis of the P3b indicated that NE phasic activity is reflected in P3b amplitudes [32]. Given that the LC is the primary source of the brain’s NE [32], it follows that stimulation of this region would enhance NE activity.

We report an effect of taVNS on P3 amplitudes during a decision-based oddball variant. The parietal P3 has links to the LC-NE system [32]. NE has also been suggested as a neuromodulator of response selection and decision-making processes, similar to those used during our Bayesian oddball task [44]. This is due to the role of NE in attentional allocation [73,74,75]. Given the links between decision-making, the parietal P3 and the NE system, our results appear to support the claim that taVNS influences NE. However, this cannot be directly inferred from electrophysiological data and remains speculative. It is also possible that other neurotransmitters are influenced by taVNS (e.g., GABA) [76]. Future studies should attempt to replicate these findings in combination with measures of NE brain concentration and functional activity levels. Nevertheless, our data support the claim of taVNS as a valid instrument of brain stimulation, despite the lack of clarity surrounding its mechanisms.

The potential clinical uses of taVNS depend on the consequences of this stimulation. The P3 is linked to attention which is demonstrated in clinical settings in studies of patients with lost consciousness [77,78]. This is also indicated in studies of attentional-deficit hyperactive disorder (ADHD), where P3 amplitudes are reduced in patients compared to controls [79]. Invasive VNS has already shown to influence neural aspects of attention in epilepsy [39]. If taVNS does reliably influence the P3, this may suggest that stimulation can influence attention [80]. The efficacy of taVNS as a method of clinical neurorehabilitation is currently being investigated in depression [81,82] and schizophrenia [83]. Both depression and schizophrenia have shown impaired attentional processes [84,85]. Other altered attentional states may therefore benefit from the use of taVNS therapy in the future, such as ADHD or generalised anxiety disorder (GAD) [86,87]. If the effects of taVNS prove effective in the context of clinical neurorehabilitation, its affordable and portable qualities could prove valuable for various patient groups during everyday life. Development of an alternative treatment could eliminate the need for invasive VNS surgeries or other pharmacological therapies. It is therefore important to establish whether taVNS reliably influences brain activity, and in what manner. However, if future research does reveal the viability of taVNS as a clinical instrument, further questions will arise concerning the proportion of non-responders versus responders [88,89], and potential habituation to treatment [63].

In addition to its possible clinical applications, taVNS has potential in a laboratory setting. If established as a non-invasive tool of neuromodulation, it would facilitate further studies with concurrent EEG recordings. These studies would require shorter timeframes and smaller budgets than typical neuromodulatory investigations.

Despite of the implications derived from our results, the study is not exempt of limitations. Our study includes a large gender imbalance, due to recruitment from a female-dominated field. Previous studies have shown gender-specific latency and amplitude differences in the P3b on an oddball task [90,91], although our study employs a repeated measures design and demonstrates internal consistency. However, it may not be applicable to the general population. Future studies may aim for a more heterogenous sample.

Further research might investigate additional physiological markers of VNS, such as pupil dilation, or salivary alpha amylase in relation to behavioral tasks related to NE. Future ERP research might join efforts to draw conclusions using specific tasks required to detect the brain stimulation effects of taVNS, following up on our data and that of another study, which also reported an effect of taVNS on oddball P3 amplitudes during particular circumstances of an oddball variant [41].

## 5. Conclusions

From our electrophysiological data, we conclude that taVNS influences parietally distributed P3 amplitudes. However, these effects seem to appear only under particular circumstances. In our data, this effect is observed during the Lake Conclusion at Draw 2. A link between taVNS and the LC-NE system has previously been suggested. This is supported on a rudimentary level by our findings that taVNS alters neural processing during a decision-making task. We conclude that taVNS probably provides effective, yet highly specific brain stimulation, pending the replicability of our results; though the supposed link between taVNS and the LC-NE system still remains an open question.

## Appendix A

**Table A1:** Randomisation of taVNS stimulation condition across sessions and distribution of the balanced versions of the Bayesian oddball task.

Subject	Active taVNS in First Session	Version of Bayesian Oddball Task			
		Red DL, Left	Yellow DL, Left	Red DL, Right	Yellow DL, Right
1	✔	✔			
2		✔			
3	✔		✔		
4				✔	
5	✔				✔
6		✔			
7	✔		✔		
8				✔	
9	✔				✔
10		✔			
11	✔		✔		
12				✔	
13	✔			✔	
14		✔			
15	✔		✔		
16				✔	
17	✔				✔
18		✔			
19	✔		✔		
20				✔	
21	✔				✔
22		✔			
23	✔		✔		
24				✔	
25	✔				✔
26		✔			
27	✔		✔		
28		✔			
29	✔				✔
30				✔	
31	✔		✔		
32				✔	
33	✔				✔
34		✔			
35	✔		✔		
36				✔	
37	✔				✔
38		✔			
39	✔		✔		
40				✔	
41	✔				✔
42		✔			
43	✔		✔		
44				✔	
45	✔				✔
46		✔			
47	✔		✔		
**Total**	20	11	11	11	8

Subjects marked in grey have been excluded. *N* = 42 included in final analysis. *N* = 20 Active First/*N* = 22 Sham First.

## Appendix B

Given that 2 stimuli were possible at each of the 3 Draws, there were a total of 8 Sequences. The probabilistic distribution (and therefore frequency) of each of these Sequences can be found in Table A2, along with the percentage probability of the SL following each Draw.

The most frequent sequences (e.g., DLF-DLF-DLF) have low variance and the sequences which have greater variance in the responses are only present in low numbers, due to their probability of occurrence (e.g., SLF-DLF-SLF).

**Table A2:** Percentage occurrence (% occurrence) of each stimulus at each Draw during the Bayesian oddball task [40]. Also provided is the posterior probability of the SL after each Draw i.e., the probability that the SL is the randomly selected lake, after witnessing the most recent Draw. According to standard Bayesian notation, this is expressed as the probability of the SL following a particular event (e) i.e., P(SL/e). For example, P(SL/DLF) = 0.03 and the P(SL/DLF-DLF) <0.01. As the task was administered twice, these probabilities naturally remained the same for each testing session. Inferential statistics were not performed on these data.

Draw 1	% Occurrence	P(SL)/e	Draw 2	% Occurrence	P(SL)/e	Draw 3	% Occurrence	P(SL)/e
DLF	0.65	0.03	DLF	0.60	<0.01	DLF	0.57	<0.01
						SLF	0.03	0.03
			SLF	0.05	0.33	DLF	0.03	0.03
						SLF	0.02	0.90
SLF	0.35	0.90	DLF	0.05	0.33	DLF	0.03	0.03
						SLF	0.02	0.90
			SLF	0.30	0.99	DLF	0.02	0.90
						SLF	0.29	0.99

## Appendix C

**Table A3:** Numbers of stimuli available for ERP analyses for each participant.

Subjects	Active						Sham					
	Draw 1		Draw 2		Draw 3		Draw 1		Draw 2		Draw 3	
	S	T	S	T	S	T	S	T	S	T	S	T
**1**	-	-	-	-	-	-	-	-	-	-	-	-
**2**	127	71	126	65	125	72	125	70	125	64	123	71
**3**	130	74	129	69	129	75	128	72	128	66	128	72
**4**	127	72	126	64	126	68	128	72	123	67	125	67
**5**	126	72	125	66	126	69	115	67	115	59	119	65
**6**	127	72	126	64	115	52	126	72	126	66	123	68
**7**	127	71	128	65	122	70	128	72	127	65	112	64
**8**	128	71	128	65	127	72	128	72	127	66	127	72
**9**	126	71	126	65	122	70	121	66	119	60	117	66
**10**	125	71	123	65	122	71	128	72	128	66	128	72
**11**	128	72	128	64	128	71	127	71	124	66	123	70
**12**	117	66	118	60	105	65	121	72	121	66	126	70
**13**	127	70	127	65	127	70	128	72	126	66	124	71
**14**	125	72	127	66	126	70	124	70	126	64	125	69
**15**	124	72	125	66	125	71	127	71	126	66	126	72
**16**	46	26	53	22	40	16	127	71	127	66	126	72
**17**	127	72	128	66	125	68	-	-	-	-	-	-
**18**	84	50	91	44	86	49	127	71	121	61	120	72
**19**	127	72	128	66	128	71	124	72	123	66	126	71
**20**	126	71	126	65	128	72	37	20	30	14	35	16
**21**	128	69	128	65	127	71	128	72	124	66	124	72
**22**	124	69	113	54	114	69	128	72	123	65	119	70
**23**	128	72	128	66	127	69	127	71	127	66	128	70
**24**	123	72	124	65	123	70	127	70	127	65	126	71
**25**	124	69	125	65	127	70	127	72	125	65	126	71
**26**	126	71	127	64	127	70	92	55	116	59	117	69
**27**	128	72	128	66	128	72	128	72	123	66	122	69
**28**	128	72	128	66	128	72	128	72	128	66	128	72
**29**	127	69	124	61	119	67	128	72	124	66	121	69
**30**	125	70	127	66	125	70	123	67	123	62	122	70
**31**	128	69	128	65	126	71	127	72	126	66	124	71
**32**	125	72	127	66	126	71	127	72	127	66	125	70
**33**	127	71	127	65	127	72	127	72	126	66	126	71
**34**	128	72	112	58	120	63	123	72	115	62	121	65
**35**	128	72	128	64	125	70	128	71	125	65	125	70
**36**	128	72	124	66	123	71	127	71	125	65	127	69
**37**	120	69	122	63	123	69	121	67	121	62	124	68
**38**	125	72	123	64	118	69	127	71	128	64	128	71
**39**	128	72	124	64	119	68	128	72	126	66	118	69
**40**	112	61	114	61	112	63	118	65	115	62	102	64
**41**	-	-	-	-	-	-	-	-	-	-	-	-
**42**	-	-	-	-	-	-	-	-	-	-	-	-
**43**	-	-	-	-	-	-	-	-	-	-	-	-
**44**	125	71	122	65	117	68	127	72	127	66	128	72
**45**	126	70	128	66	127	71	128	72	128	66	127	72
**46**	115	64	114	59	112	66	127	72	128	66	128	72
**47**	126	69	123	61	120	68	122	68	107	52	112	57
**Total:**	**128**	**72**	**128**	**72**	**128**	**72**	**128**	**72**	**128**	**72**	**128**	**72**

## Appendix D

### Appendix D.1. %. DLS

The mean %DLS of each stimulus, separated by Draw and taVNS condition, is shown in Appendix A Figure A1. The variance on the most common trials (DLF-DLF-DLF and SLF-SLF-SLF) is close to zero. These trials were more frequent due to the probability distribution of the lakes and offered greater certainty about whether the DL or SL was more likely. That is to say, a DLF in the first draw resulted in the participants selecting the DL 98.8% of the time (*SE* = 0.49).

On closer inspection of the data, Sequences which require an alternating %DLS (DLF-SLF-SLF, SLF-DLF-DLF) account for less than 5% of overall trials. A %DLS of either >99% or <1% is reached in the majority of trials (DLF-DLF-DLF and SLF-SLF-SLF). In over 95% of the sequences presented, the participants reached a “Lake Conclusion” at Draw 2. From these descriptive data, it appears that subjects did not change their decision following their choice at Draw 2. The stimuli presented at Draw 3 were then inconsequential for the following selections. This is also consistent with the Bayesian expectations.

### Appendix D.2. Reaction Times

Reaction Times (RTs) were calculated as the mean value of individual medians. Overall mean RTs for each draw of each sequence were calculated. Stimuli for which the RT was less than 100 ms or greater than 3 SDs above the individual median were excluded from analysis. One additional subject was removed on the grounds that 40% of stimuli in the sham condition were excluded for these reasons. This resulted in *N =* 41 for the behavioural data analysis. Overall mean RTs are presented in Table A4. However, participants were instructed to pay more attention to accurate decisions, rather than fast responses. Thus, this information is purely for descriptive purposes.

**Table A4:** Overall mean reaction times (RT) for each stimulus (after exclusion), calculated from individual medians. These have been separated by active and sham taVNS conditions. Reaction times are not separated according to %DLS. N indicates the number of subjects that had available data for these sequences. Inferential statistics were not performed on these data.

ACTIVE								
Draw 1	*M*	*SD*	Draw 2	*M*	*SD*	Draw 3	*M*	*SD*
DLF	496.6	115.0	DLF	325.3	88.5	DLF	293.3	96.8
						SLF	471.8	299.7
			SLF*	616.5	334.6	DLF *	557.1	140.3
						SLF	585.8	228.2
SLF	594.9	141.7	DLF*	682.4	318.4	DLF *	558.2	246.1
						SLF	560.1	205.0
			SLF	369.8	96.1	DLF *	537.7	385.7
						SLF	291.0	94.8
SHAM								
Draw 1	*M*	*SD*	Draw 2	*M*	*SD*	Draw 3	*M*	*SD*
DLF	498.3	126.1	DLF	317.4	67.0	DLF	325.3	88.5
						SLF	595.0	141.7
			SLF	663.3	401.1	DLF	496.6	115.0
						SLF	574.1	206.7
SLF	583.7	170.8	DLF	712.0	380.7	DLF	369.8	96.1
						SLF	533.8	178.0
			SLF	366.3	85.9	DLF	468.7	386.5
						SLF	295.5	85.6

* These sequences have data from only *N* = 40 subjects. One subject’s RT were excluded due to being less than 100 ms or greater than 3 SDs above their personal mean.

## Appendix E

Inferential statistical analysis of RIDE-corrected data for additional time windows.

### Appendix E.1. 300–349 ms

#### Appendix E.1.1. Draw 1

A typical main effect of Stimulus was found (*F*(1,41) = 14.28, *p* < 0.01, η*_p_*^2^ = 0.26), but taVNS did not show a significant main effect (*F*(1,41) = 0.14, *p* = 0.72, η*_p_*^2^ < 0.01) or interaction with Stimulus (*F*(1,41) = 0.25, *p* = 0.62, η*_p_*^2^ = 0.01).

#### Appendix E.1.2. Draw 2

The main effect of Stimulus was significant again at Draw 2 (*F*(1,41) = 18.19, *p* < 0.01, η*_p_*^2^ = 0.31). The main effect of taVNS was not significant (*F*(1,41) = 0.58, *p* = 0.45, η*_p_*^2^ = 0.01). However, the taVNS × Stimulus interaction did not show statistical significance (*F*(1,41) = 0.58, *p* = 0.45, η*_p_*^2^ = 0.01).

#### Appendix E.1.3. Draw 3

No main effect of Stimulus or taVNS was present at Draw 3 (Stimulus: *F*(1,41) = 0.29, *p* = 0.59, η*_p_*^2^ = 0.01; taVNS: *F*(1,41) = 2.05, *p* = 0.16, η*_p_*^2^ = 0.05). The interaction between taVNS and Stimulus was not statistically significant (*F*(1,41) = 0.01, *p* = 0.92, η*_p_*^2^ < 0.01).

### Appendix E.2. 350–399 ms

#### Appendix E.2.1. Draw 1

A typical main effect of Stimulus was found (*F*(1,41) = 29.21, *p* < 0.01, η*_p_*^2^ = 0.42) but taVNS did not show a significant main effect (*F*(1,41) = 0.36, *p* = 0.55, η*_p_*^2^ = 0.01) or interaction with Stimulus (*F*(1,41) = 0.16, *p* = 0.69, η*_p_*^2^ < 0.01).

#### Appendix E.2.2. Draw 2

The main effect of Stimulus was significant again at Draw 2 (*F*(1,41) = 35.12, *p* < 0.01, η*_p_*^2^ = 0.46). The main effect of taVNS was not significant (*F*(1,41) = 0.20, *p* = 0.66, η*_p_*^2^ = 0.01), nor was the taVNS × Stimulus interaction (*F*(1,41) = 4.03, *p* = 0.05, η*_p_*^2^ = 0.09).

#### Appendix E.2.3. Draw 3

No main effect of Stimulus or taVNS was present at Draw 3 (Stimulus: *F*(1,41) = 2.19, *p* = 0.15, η*_p_*^2^ = 0.05; taVNS: *F*(1,41) = 1.77, *p* = 0.19, η*_p_*^2^ = 0.04). The interaction between taVNS and Stimulus was not statistically significant (*F*(1,41) = 0.23, *p* = 0.63, η*_p_*^2^ < 0.01).

### Appendix E.3. 450–499 ms

#### Appendix E.3.1. Draw 1

A typical main effect of Stimulus was found (*F*(1,41) = 19.57, *p* < 0.01, η*_p_*^2^ = 0.32) but taVNS did not show a significant main effect (*F*(1,41) = 0.21, *p* = 0.65, η*_p_*^2^ = 0.01) or interaction with Stimulus (*F*(1,41) = 0.04, *p* < 0.83, η*_p_*^2^ < 0.01).

#### Appendix E.3.2. Draw 2

The main effect of Stimulus was significant again at Draw 2 (*F*(1,41) = 20.34, *p* < 0.01, η*_p_*^2^ = 0.33). The main effect of taVNS was not significant (*F*(1,41) = 0.23, *p* = 0.64, η*_p_*^2^ = 0.01). However, the taVNS × Stimulus interaction was not statistically significant (*F*(1,41) = 3.78, *p* = 0.06, η*_p_*^2^ = 0.08).

#### Appendix E.3.3. Draw 3

No main effect of Stimulus or taVNS was present at Draw 3 (Stimulus: *F*(1,41) = 1.02, *p* = 0.32, η*_p_*^2^ = 0.02; taVNS: *F*(1,41) = 0.93, *p* = 0.34, η*_p_*^2^ = 0.02). The interaction between taVNS and Stimulus was not statistically significant (*F*(1,41) = 0.05, *p* = 0.83, η*_p_*^2^ < 0.01).
